# Differentiation of T Helper 17 Cells May Mediate the Abnormal Humoral Immunity in IgA Nephropathy and Inflammatory Bowel Disease Based on Shared Genetic Effects

**DOI:** 10.3389/fimmu.2022.916934

**Published:** 2022-06-13

**Authors:** Jianbo Qing, Changqun Li, Xueli Hu, Wenzhu Song, Hasna Tirichen, Hasnaa Yaigoub, Yafeng Li

**Affiliations:** ^1^ The Fifth Clinical Medical College of Shanxi Medical University, Taiyuan, China; ^2^ Department of Nephrology, Shanxi Provincial People’s Hospital (Fifth Hospital) of Shanxi Medical University, Taiyuan, China; ^3^ School of Public Health, Shanxi Medical University, Taiyuan, China; ^4^ Institutes of Biomedical Sciences, Shanxi University, Taiyuan, China; ^5^ Core Laboratory, Shanxi Provincial People’s Hospital (Fifth Hospital) of Shanxi Medical University, Taiyuan, China; ^6^ Shanxi Provincial Key Laboratory of Kidney Disease, Taiyuan, China; ^7^ Academy of Microbial Ecology, Shanxi Medical University, Taiyuan, China

**Keywords:** IgA nephropathy, inflammatory bowel disease, genetic effects, mucosal immune, T helper 17 cell

## Abstract

**Background:**

IgA nephropathy (IgAN) is the most frequent glomerulonephritis in inflammatory bowel disease (IBD). However, the inter-relational mechanisms between them are still unclear. This study aimed to explore the shared gene effects and potential immune mechanisms in IgAN and IBD.

**Methods:**

The microarray data of IgAN and IBD in the Gene Expression Omnibus (GEO) database were downloaded. The differential expression analysis was used to identify the shared differentially expressed genes (SDEGs). Besides, the shared transcription factors (TFs) and microRNAs (miRNAs) in IgAN and IBD were screened using humanTFDB, HMDD, ENCODE, JASPAR, and ChEA databases. Moreover, weighted gene co-expression network analysis (WGCNA) was used to identify the shared immune-related genes (SIRGs) related to IgAN and IBD, and R software package org.hs.eg.db (Version3.1.0) were used to identify common immune pathways in IgAN and IBD.

**Results:**

In this study, 64 SDEGs and 28 SIRGs were identified, and the area under the receiver operating characteristic curve (ROC) of 64 SDEGs was calculated and two genes (MVP, PDXK) with high area under the curve (AUC) in both IgAN and IBD were screened out as potential diagnostic biomarkers. We then screened 3 shared TFs (SRY, MEF2D and SREBF1) and 3 miRNAs (hsa-miR-146, hsa-miR-21 and hsa-miR-320), and further found that the immune pathways of 64SDEGs, 28SIRGs and 3miRNAs were mainly including B cell receptor signaling pathway, FcγR-mediated phagocytosis, IL-17 signaling pathway, toll-like receptor signaling pathway, TNF signaling pathway, TRP channels, T cell receptor signaling pathway, Th17 cell differentiation, and cytokine-cytokine receptor interaction.

**Conclusion:**

Our work revealed the differentiation of Th17 cells may mediate the abnormal humoral immunity in IgAN and IBD patients and identified novel gene candidates that could be used as biomarkers or potential therapeutic targets.

## Introduction

IgA nephropathy (IgAN), which could be caused by primary or secondary diseases (1), was first described by Berger in 1968 as a group of glomerular diseases characterized by mesangial hyperplasia and significantly diffuse IgA deposits in the mesangium (2). IgAN has distinct geographical and ethnic differences, it is more common in Asian populations than in Caucasians (3). Recently, IgAN has attracted attention as a major renal manifestation of inflammatory bowel disease (IBD), which is characterized by chronic inflammation of the gastrointestinal tract and mainly contains Crohn’s disease (CD) and ulcerative colitis (UC) (4).

By studying the gut-kidney axis and gut immunity, several studies have shown that presentation and exacerbation of IgAN coincide with IBD activity (5). Localized gastrointestinal immunosuppression as an important treatment of primary IgAN likewise verifies the pathogenic role of gut immune in the development and progression of IgAN (6). Additionally, genome-wide association studies (GWAS) have shown some significant genome-wide associations in IgAN and IBD. The identified risk genes are associated with abnormal intestinal immunity, which could increase susceptibility to IgAN and IBD as a shared genetic risk (7, 8). However, current research findings on the genetic effects shared by IgAN and IBD are not abundant, which has hindered the improvement of prevention and treatment strategies for these diseases.

We aimed to focus on the genetic effects and abnormal immunity in IgAN and IBD. The differential expression analysis and weighted gene co-expression network analysis (WGCNA) were conducted on gene expression data of IgAN, UC and CD, which were obtained from the Gene Expression Omnibus (GEO) (9). We identified 64 shared differentially expressed genes (SDEGs) and 28 shared immune-related genes (SIRGs) and found that CD has more similar genetic effects with IgAN than UC. In addition, shared transcription factors (TFs) including SRY, MEF2D, SREBF1, and miRNAs (hsa-miR-146, hsa-miR-21 and hsa-miR-320) were explored based on multiple databases. According to the enrichment analysis of SEDGs, SIRGs, and miRNAs, shared genetic effects mainly affect IgAN and IBD through the abnormal humoral immunity mediated by Th17 cells, as well as TRP channels.

## Methods

### Microarray Data Download and Process

The key word “IgA Nephropathy”, “Ulcerative colitis”, and “Crohn’s disease” were used to search IgAN and IBD gene expression profiles in the GEO database. For accuracy and reliability, we adopted the following criteria filter: 1. IgAN and IBD sequencing results should be obtained from the analysis of human blood samples. 2. The datasets must include cases and controls, and the number of samples in each group should be more than 6 to ensure the accuracy of the analysis. 3. The datasets must have balanced data distribution. 4. Patients had not received immunosuppressive therapy one month prior to sequencing analysis. Finally, the GEO datasets numbered GSE14795 (10) and GSE119600 (11) were selected. We first performed log2 transformation for gene expression profiling and then the probes were transformed into corresponding gene symbols under the platform annotation information. The information of the two datasets is shown in **Table 1**.

**Table 1:** The information of samples used to analysis.

Datasets	Samples	Tissue
GSE14795	12IgAN patients and 8 healthy controls	Whole blood
GSE119600	45UC patients (adults), 47CD patients (adults) and 47 healthy controls (adults)	Whole blood

IgAN, IgA Nephropathy; UC, Ulcerative colitis; CD, Crohn’s disease.

### Identification of Shared Differentially Expressed Genes

To identify the shared genetic effects of IgAN and IBD, the Limma R package (12) was used to explore the DEGs in GSE14795 and GSE119600, an adjusted “*P* value < 0.05” was set as the threshold for screening of the DEGs. Then, the DEGs of IgAN and IBD were combined into SDEGs. Additionally, logFC-logFC plots generated by GraphPad Prism 8.0.2 and dimension reduction analysis were used to demonstrate the similarity between IgAN and IBD. Finally, receiver operator characteristic (ROC) analysis was performed using the R software package pROC(Version 1.17.0.1) (13) to obtain the porch area under the receiver operating characteristic curve (AUC) for the SDEGs in IgAN and IBD.

### The Common Transcription Factors-SDEGs Network Construction

A list of 1665 TFs was downloaded from HumanTFDB (14) to investigate the transcriptionally regulated activity of SDEGs (**Supplementary Table 1**). ENCODE (14), JASPAR (15), and ChEA (16) databases were used to obtain the targeted genes of TFs and Cytoscape 3.8.2 was used to generate the TFs-SDEGs network.

### The Common miRNAs-SDEGs Network Construction

We obtained IgAN and IBD-associated miRNAs and their intersections from the Human microRNA Disease Database (HMDD), which is a database that organizes experimentally-supported evidence for human miRNAs and disease associations (17). We then determined the expression levels of these miRNAs in IgAN and IBD based on published literature. The target genes of common miRNAs were explored in miRTarbase, which is an experimentally validated miRNA-target interactions database, and it was used to obtained the targeted mRNAs (18). Meanwhile, the pathways of common miRNAs were obtained from ENCORI database (19). Finally, the intersection of target genes of common miRNAs and shared genes in IgAN and IBD were used to construct the miRNAs–mRNAs regulated network. The sankey diagram was used to visualize the network.

### The Immune-Related Pathway Enrichment Analysis of SDEGs

For function analysis of SDEG, gene annotations in R software package org.hs.eg.db (Version3.1.0) were used as background to map genes into gene sets and R software package clusterProfiler (Version3.14.3) was used for Kyoto Encyclopedia of Genes and Genomes (KEGG) pathway enrichment analysis (20), and then the immune related pathways were screened and visualized with a bubble chart.

### Weighted Gene Co-Expression Network Analysis of ImmuneRelated Genes

Abnormal autoimmunity is not only an important feature of IgAN and IBD, but also an important link between them. Thus, we attempted to explore the shared genetic effects between IgAN and IBD from another perspective. We downloaded the immune-related genes (IRGs) from the ImmPort database (https://www.immport.org/shared/) to further explore and verify the common immune mechanisms of IgAN and IBD. After eliminating duplicate genes, we finally obtained 1793 IRGs (**Supplementary Table 2**). Then, we selected the IRGs of GSE14795 and GSE119600 for the WGCNA, which is an algorithm that can find co-expression gene modules with high biological significance and explore the relationship between gene networks and diseases. Firstly, the Pearson’s correlation matrices and average linkage method were both performed for all pair-wise genes. Then the appropriate soft powers β (ranged from 1 to 20) was selected using the function of “pick Soft Threshold” in the WGCNA package. After choosing the power of β, the adjacency was transformed into a topological overlap matrix (TOM), which could measure the network connectivity of a gene defined as the sum of its adjacency with all other genes for network generation. The corresponding dissimilarity (1-TOM) was calculated, which was used to classify genes with similar expression profiles into gene modules. Each module has a correlation coefficient and *P-*value corresponding to clinical characteristics, the modules with high correlation coefficient and a *P*-value of <0.05 were selected and the genes in these modules were screened out for subsequent analyses. In this study, the soft threshold β was 8 in the WGCNA of IgAN, 4 in UC, and 2 in CD. The other parameters were the following: networkType = “unsigned”, minModuleSize = 25, R square cut = 0.85 and deepSplit = 2.

### The Function Enrichment Analysis of Shared Immune-Related Genes

The Human Phenotype Ontology (HPO, https://hpo.jax.org) is widely used in neurology, nephrology, immunology, and pulmonology, which provides a comprehensive logical standard to describe and computationally analyze phenotypic abnormalities found in human disease (21), and the function enrichment analysis of SIRGs were preformed based on HPO and KEGG using R software package clusterProfiler (Version 3.14.3), similarly, screen out the immune-related sections.

## Results

### Identification of SDEGs in IgAN and IBD

**Figure 1:**
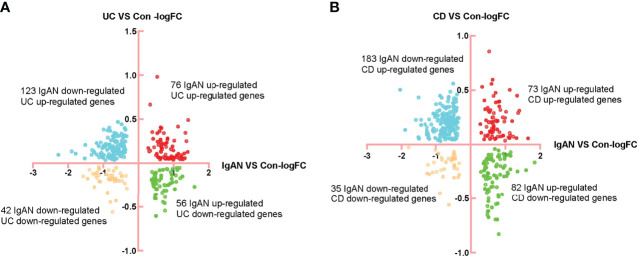
Identification of DEGs of IgAN, UC, and CD. **(A)** FC-FC plot of IgAN and UC, there were 76 common up-regulated genes and 42 common down-regulated genes in IgAN and UC. **(B)** FC-FC plot of IgAN and CD, there were 73 common up-regulated genes and 35 common down-regulated genes in IgAN and CD.

**Figure 2:**
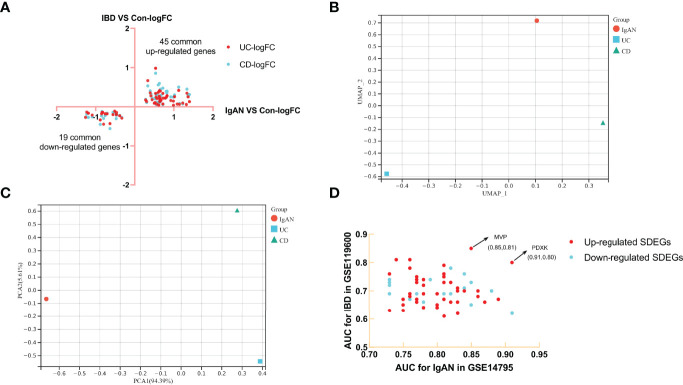
Identification of SDEGs of IgAN and CD, dimension reduction analysis and AUC calculation. **(A)** FC-FC plot of IgAN and IBD, there were 45 common up-regulated genes and 19 common down-regulated genes. **(B)** UAMP analysis of SDEGs log_2_FC in IgAN, UC, and CD. **(C)** PCA analysis of SDEGs log_2_FC in IgAN, UC, and CD, IgAN is closer to CD than IgAN is to UC in both UAMP and PCA analysis. **(D)** AUC-AUC plot of IgAN and IBD, the AUC of MVP and PDXK in IgAN and IBD were both≥0.8.

Limma R package was used to identify the DEGs in IgAN, UC and CD (**Supplementary Table 3**). There were 76 common up-regulated genes and 42 common down-regulated genes in IgAN and UC, while there were 73 common up-regulated genes and 35 common down-regulated genes in IgAN and CD (**Figures 1A, B**). Moreover, we found 45 common up-regulated genes and 19 common down-regulated genes (**Figure 2A**), which is considered as SDEGs of IgAN and IBD. The expression characteristics of SDEGs in IgAN and IBD had certain similarities. However, dimensional reduction analysis showed that CD and IgAN were closer than UC, as the distance of UC and IgAN is farther than CD and IgAN in **Figures 2B, C**, implying that CD and IgAN share more genetic effect similarities based on 64 SDEGs. Additionally, **Figure 2D** shows the AUC of 64 SDEGs in IgAN and IBD, MVP and PDXK were considered as potential diagnostic biomarkers in blood since their AUC in IgAN and IBD were both≥0.8 (**Supplementary Table 4**).

### The Common TFs-SDEGs Network Construction

**Figure 3:**
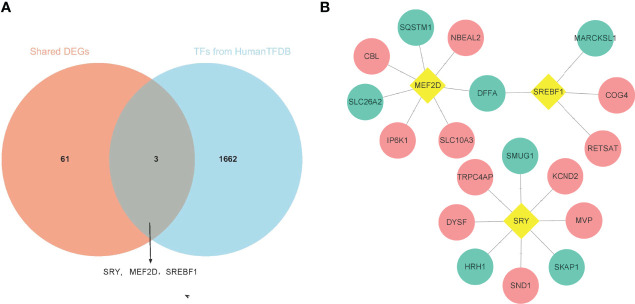
Identification of shared TFs. **(A)** The venn diagram of 64 SDEGs and 1665 TFs obtained from HumanTFDB, SRY, MEF2D, and SREBF1 were identified as shared TFs. **(B)** Network of TFs-SDEGs, the yellow rhombus represented TFs, the pink circle represented up-regulated SDEGs, and the blue circle represented the down-regulated SDEGs.

Three up-regulated TFs were identified in the SDEGs of IgAN and IBD (**Figures 3A**), and they are related to the expression of 18 SDEGs in comparison with the data of ENCODE, JASPAR and ChEA databases (**Figures 3B** and **Supplementary Table 5**). SRY, MEF2D and SREBF1 may be the hub SDEGs and shared TFs for IgAN and IBD.

### The Common miRNAs-SDEGs Network Construction

**Figure 4:**
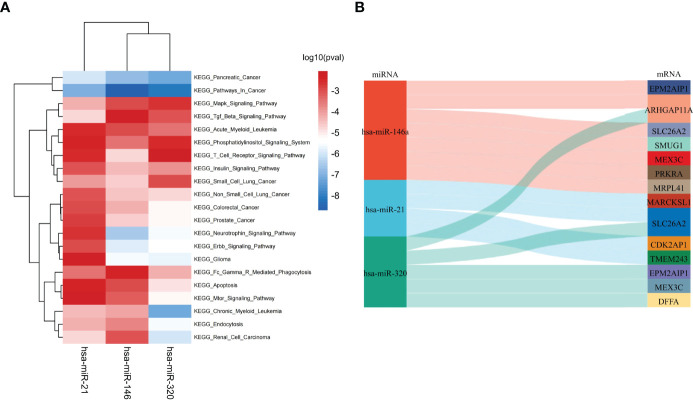
Identification of shared miRNAs. **(A)** KEGG enrichment analysis of 3 shared miRNAs (hsa-miR-146, hsa-miR-21, and hsa-miR-320). **(B)** The sankey diagram of 3 shared miRNAs and 14 down-regulated SDEGs.

According to the HMDD database, there were 4 common miRNAs between IgAN and IBD (**Supplementary Table 6**). We further screened the common miRNAs according to the published literature provided by the HMDD database, and 3 miRNAs were identified, including hsa-miR-146, hsa-miR-21, and hsa-miR-320. Then the KEGG pathway of the three miRNAs were further studied using ENCORI database (**Figure 4A**). The results showed that these miRNAs were involved in phosphatidylinositol signaling system, T cell receptor signaling pathway, and Fc gamma receptor (FcγR)-mediated phagocytosis, which were related to humoral immunity. We then obtained the targeted mRNAs of the three miRNAs and identified 14 mRNAs from 19 down-regulated SDEGs to establish the common miRNAs-mRNAs network for IgAN and IBD (**Figure 4B** and **Supplementary Table 7**), which could serve as a novel marker for the prevention and treatment of IgAN and IBD.

### The Immune-Related Pathway Enrichment Analysis of SDEGs

**Figure 5:**
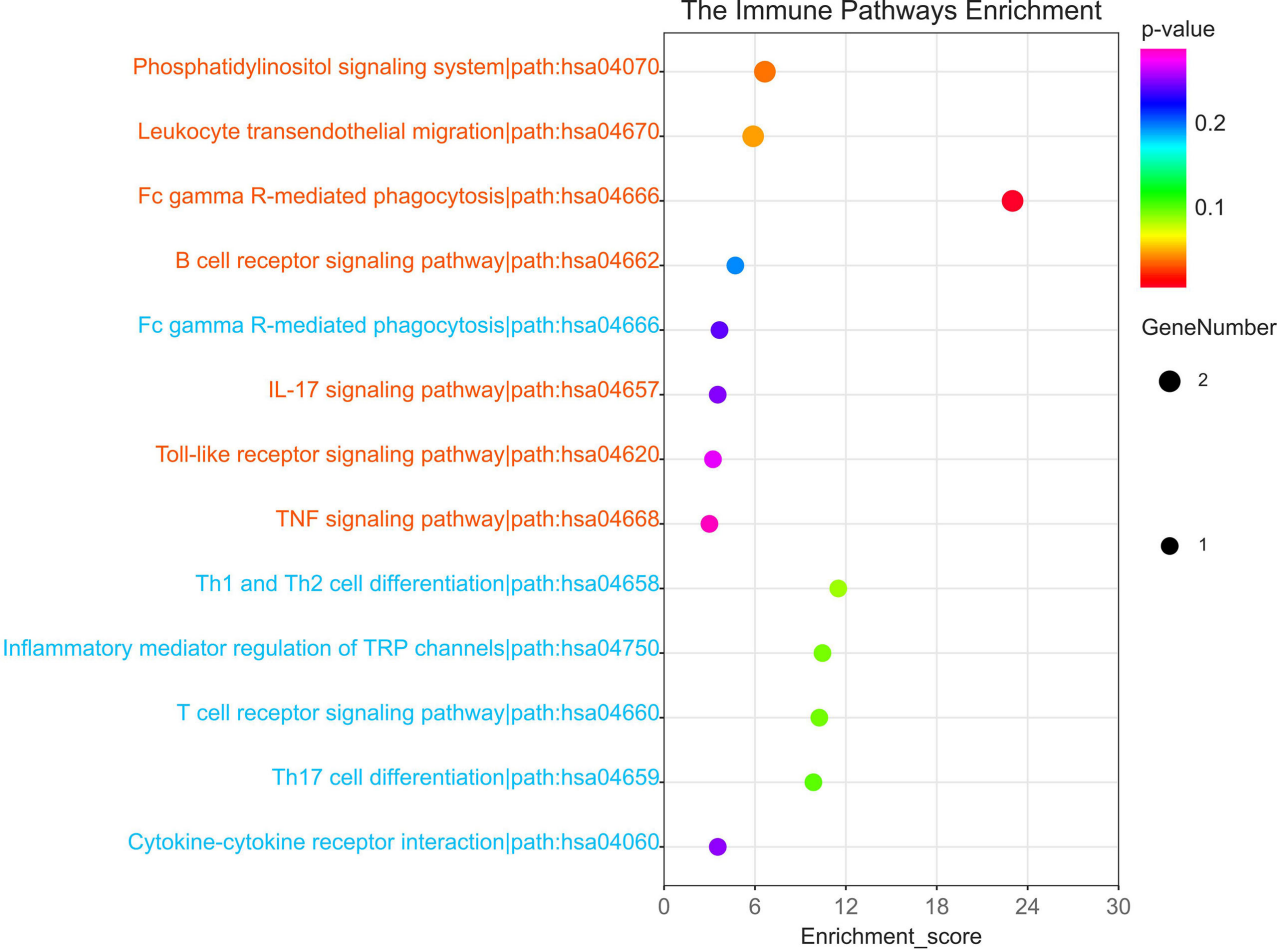
The immune-related KEGG pathway of 64SDEGs. Red colour represented the pathway in which up-regulated SDEGs were involved while blue colour represents the pathway in which down-regulated SDEGs were involved.

KEGG enrichment analysis was performed in 45 up-regulated SDEGs and 19 down-regulated SDEGs respectively. The immune-related pathways were screened and presented in **Figure 5**. The genes in each pathway were shown in **Table 2**. 5 up-regulated genes were involved in phosphatidylinositol signaling system, leukocyte transendothelial migration, B cell receptor signaling pathway, FcγR-mediated phagocytosis, IL-17 signaling pathway, toll-like receptor signaling pathway, and TNF signaling pathway. INPPL1 gene was closely related to the activation of B cells, while MMP gene was associated with the function of IL-17 and TNF. Additionally, 4 down-regulated SDEGs were related to FcγR-mediated phagocytosis, Th1 and Th2 cell differentiation, inflammatory mediator regulation of TRP channels, T cell receptor signaling pathway, Th17 cell differentiation, and cytokine-cytokine receptor interaction. Most noteworthy was that the decrease of CD4 which promotes the differentiation of Th17 cell while inhibiting the differentiation of Th1 and Th2 cell (**Supplementary Figure 1** and **2**). The above results indicate a similar abnormal humoral immunity in IgAN and IBD, which may be a common pathogenesis and therapeutic target.

**Table 2:** The genes in the immune-related pathways.

	Term description	Gene Symbols
Up-regulated SDEGs	Phosphatidylinositol signaling system	IP6K1;INPPL1
	Leukocyte transendothelial migration	THY1;MMP9
	B cell receptor signaling pathway	INPPL1
	Fc gamma R-mediated phagocytosis	INPPL1
	IL-17 signaling pathway	MMP9
	Toll-like receptor signaling pathway	TLR6
	TNF signaling pathway	MMP9
Down-regulated SDEGs	Fc gamma R-mediated phagocytosis	PIP5K1B;MARCKSL1
	Th1 and Th2 cell differentiation	CD4
	Inflammatory mediator regulation of TRP channels	HRH1
	T cell receptor signaling pathway	CD4
	Th17 cell differentiation	CD4
	Cytokine-cytokine receptor interaction	CD4

### Identification of SIRGs in IgAN and IBD

**Figure 6:**
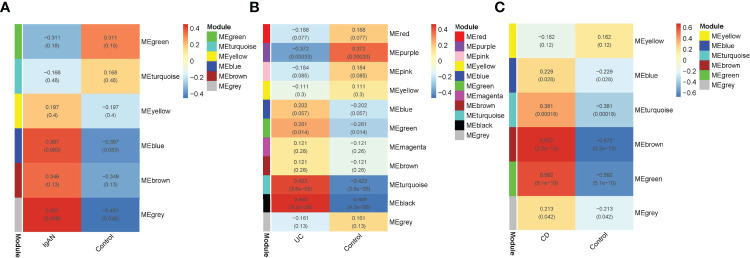
WGCNA analysis of IRGs. **(A)**: Module–trait relationships in IgAN. Each cell contains the corresponding correlation and -value. **(B)**: Module–trait relationships in UC. Each cell contains the corresponding correlation and *p*-value. **(C)**: Module–trait relationships in CD. Each cell contains the corresponding correlation and *p*-value. Modules with R > 0.4 and *P < *0.05 were screened for further study.

**Figure 7:**
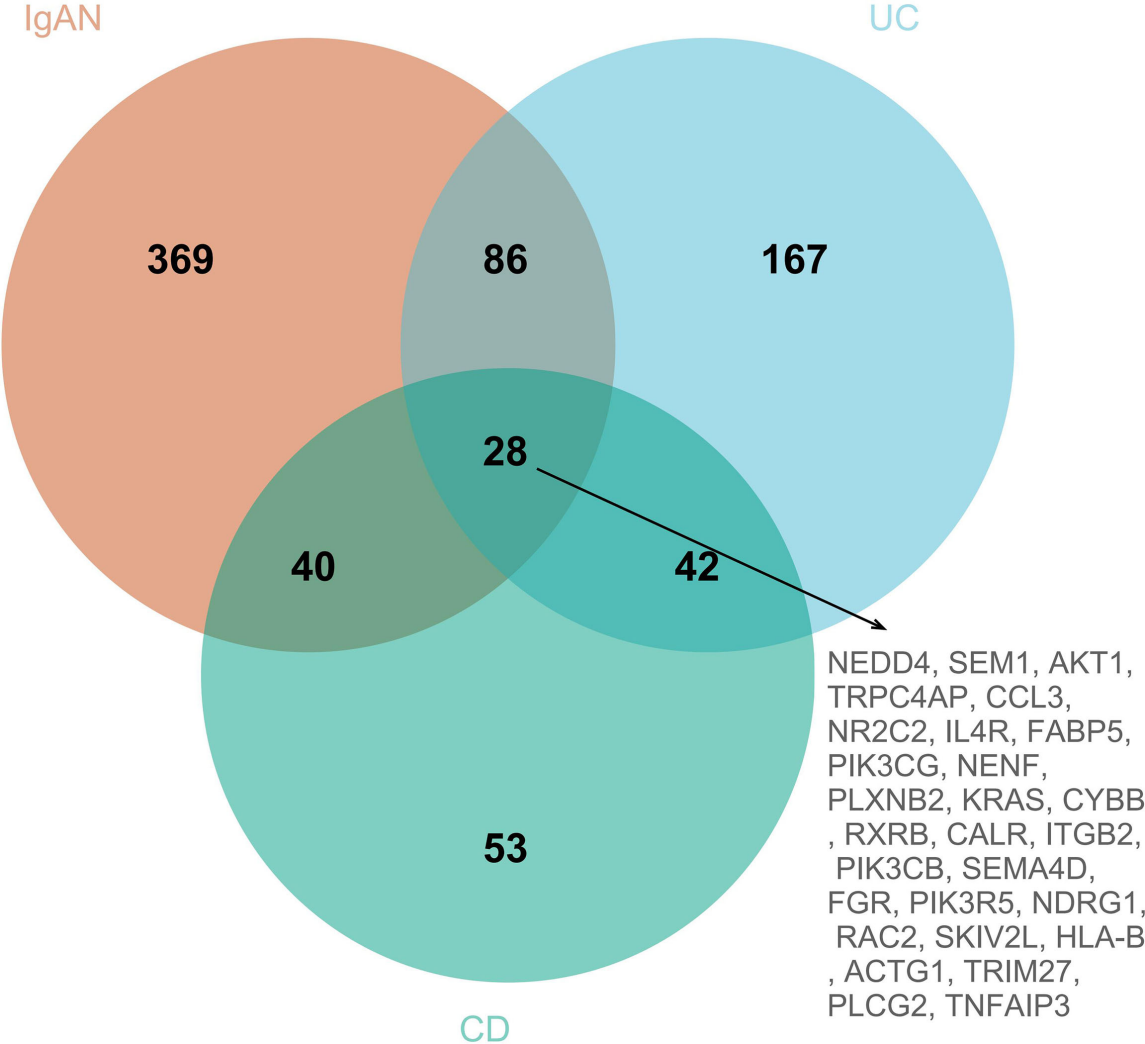
The venn diagram of SIRGs, 28 SIRGs were identified form grey module in IgAN, turquoise and black modules in UC, and brown and green modules in CD.

To explore the IRGs that were positively associated with IgAN and IBD, 5 modules in IgAN, 11 modules in UC, and 6 modules in CD were identified using WGCAN. The spearman correlation coefficient and *P* value between each module and disease were shown in **Figure 6**. Grey module in IgAN, turquoise and black modules in UC, brown and green modules in CD were selected for further study according to R>0.4 and *P<*0.05, respectively. The gray module was positively correlated with IgAN, including 523 IRGs. Similarly, the turquoise and black modules were positively correlated with UC, including 323 IRGs, while there were 163 IRGs which were positively correlated with CD in brown and green modules of CD (**Supplementary Table 8**). The results of the three diseases could be combined to get the shared IRGs of IgAN and IBD. Finally, 28 SIRGs which were positively correlated with both IgAN and IBD were identified (**Figure 7**).

### The Enrichment Analysis of SIRGs

**Figure 8:**
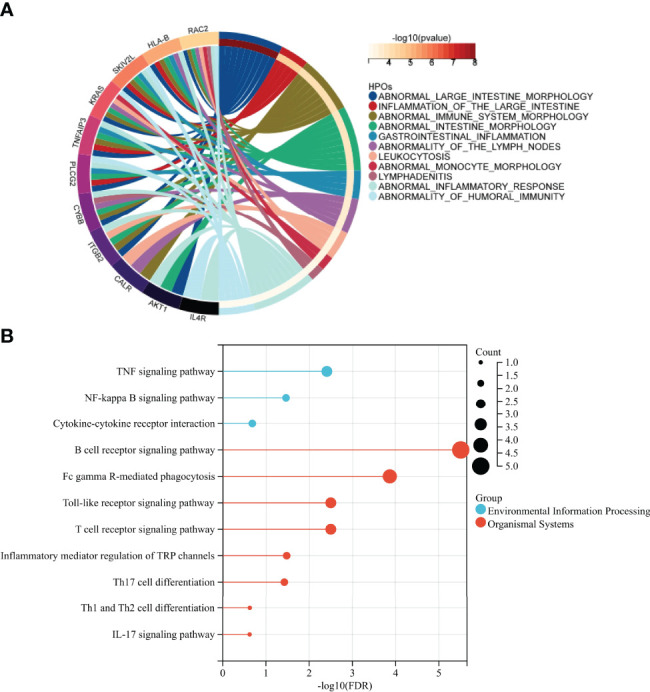
Identification of the immune function of SIRGs **(A)** HPO enrichment analysis showed that 28 SIRGs were associated with abnormal intestinal immunity. **(B)** The immune related KEGG pathway of 28SIRGs.

The results of HPO enrichment analysis indicated that 28 SIRGs were closely associated with abnormal intestinal morphology and intestinal immunity, especially humoral immunity (**Figure 8A**). Besides, the immune-related pathways analysis based on KEGG showed that the 28 SIRGs play an important role in TNF signaling pathway, NF-kappa B signaling pathway, cytokine-cytokine receptor interaction, B cell receptor signaling pathway, FcγR-mediated phagocytosis, toll-like receptor signaling pathway, T cell receptor signaling pathway, inflammatory mediator regulation of TRP channels, Th17 cell differentiation, Th1 and Th2 cell differentiation, and IL-17 signaling pathway, which confirms our findings in previous sections that abnormal differentiation of T cell and abnormal activation of B cell resulted in abnormal humoral immunity shared by IgAN and IBD (**Figure 8B**).

## Discussion

Intestinal mucosa has the largest population of immune cells in the human body (8), and the mucosal immunity has been considered to play a critical role in the pathogenesis of IgAN (22). IgAN is the most frequent glomerulonephritis in IBD and has a significantly higher diagnostic prevalence than all non-IBD renal biopsies (23), which indicates that there may be shared pathogenic mechanisms and therapeutic targets between them.

### Novel Biomarkers for IgAN and IBD

As a potential risk factor of renal insufficiency development in patients with IBD (24), the diagnosis of IgAN mainly relies on pathological biopsy (25, 26). However, the acceptance rate and execution rate of renal biopsy are not sufficient. Thus, early diagnosis of IgAN is crucial to protect renal function in patients with IBD (27).

64 SDEGs were screened through differential analysis of microarray data of IgAN, UC, and CD patients. Dimension reduction analysis indicated that the expression of SDEGs in CD is closer to IgAN than to UC, which suggests there may be more genetic effect similarities between CD and IgAN. A cross-sectional analysis also showed that the frequency of IgAN was significantly higher in patients with CD (11/207, 5.3%) than in those with UC (2/220, 0.9%) (28), this may be related to more inflammatory infiltration and paneth cells in the ileum than colorectum (29). In addition, MVP and PDXK with higher AUC in 64SDEGs were identified as potential biomarkers in the blood of IgAN and IBD, which may facilitate the diagnosis of these diseases.

We then further identified 3 TFs, including SRY, MEF2D, and SREBF1, based on 64 SDEGs and HumanTFDB. These TFs were up-regulated in IgAN and IBD and were correlated with 18 SDEGs. TFs could act both as activators and repressors of gene expression at the transcriptional level (29), changing the expression of the unique TFs to treat IgAN and IBD may be more effective with less effort. Moreover, three miRNAs (hsa-miR-146, hsa-miR-21 and hsa-miR-320) were screened using HMDD, which could down-regulate the expression of 14 SDEGs at the posttranscriptional level and play an important role in the pathophysiology of IgAN and IBD (30).

Additionally, we screened 28 SIRGs that were positively correlated with IgAN and IBD using WGCNA analysis. The occurrence and development of IgAN and IBD are dominated by immunity, and HLA-DR1 (31), which is one of the IRGs, has been investigated and described as a genetic susceptibility in both IgAN and IBD. The discovery of SIRGs would be beneficial to explore the shared pathogenesis between IgAN and IBD and provide novel therapeutic targets.

### Abnormal Humoral Immunity Mediated by Th17 Cells in IgAN and IBD

The underlying mechanism and inciting agent of IgAN and IBD is a complex and unclear, but many researches have shown that T cell-mediated mucosal immunity is essential in the pathogenesis of IgAN and IBD (32, 33). The IgA produced by the intestinal mucosal barrier in humoral immune response to infection or other stimulation forms, a circulating immune complex which is deposited in the kidney resulting in renal insufficiency with the release of cytokines and growth factors (34).

We identified 64 SDEGs and 28 SIRGs by limma and WGCNA analysis. SDEGs were based on the perspective of gene differential expression, while SIRGS were based on the correlation with diseases. Although SDEGs and SIRGs were quite different, they were involved in same immune pathways that mainly related to the differentiation of T cells and the activation of B cells.

The enrichment analysis showed that the changes of SDEGs, miRNAs, and SIRGs were related to T cell receptor (TCR) pathway. TCR could mediate recognition of antigenic peptides bound to MHC molecules and lead to the activation and differentiation of CD4+ T cell (35). It is worth noting that SDEGs and SIRGs were related to the differentiation of Th17 cell, which cause autoimmunity and inflammation (36). Th17 cell could promote the immune response and activate various inflammatory pathways through the IL-17 signaling pathway (37). Infection and other factors can up-regulate the expression of Th17 cell and Th17-related cytokines in mucosal immunity, thereby inducing or aggravating IgAN and IBD (38, 39). The shared miRNAs were involved in TGF-beta signaling pathway, which is dispensable for generation of pathogenic Th17 cell. miRNAs may promote this biological process by down-regulating the expression of related genes. Th17 cell and regulatory T cell(Treg) play a crucial role in the regulation of immune response in mucosal immunity (40), the Th17/Treg immune balance was not only involved in the occurrence of IgAN (41), but also related to the pathogenesis of IBD. Th 17 cells and Treg cells, which were differentiated from CD4+ T cells, promote tissue inflammation and suppress autoimmunity respectively (39), understanding and regulating the balance between Th17/Treg cells would contribute to the treatment and prevention of IgAN and IBD.

In addition, phosphatidylinositol 3 kinase (PI3K) and phospholipase C-Gamma 2 (PLC-Gamma 2) are important downstream effectors of BCR signaling, which ultimately lead to the activation of B cell proliferation, differentiation, and antibody production (42). The activation of phosphatidylinositol signaling system represents the massive activation of B cell and the secretion of antibodies in IgAN and IBD. This was confirmed by functional enrichment analysis of SEDGs, SIRGs and shared miRNAs. IL-17 or toll-like receptor stimulates the proliferation of B cells and induces the dysglycation of IgA1, which eventually leads to IgAN (43, 44). Besides, FcγR, which are cell surface glycoproteins that mediate cellular effector functions of IgG antibodies, can influence susceptibility to a variety of antibody-mediated intestinal inflammatory and autoimmune (45). FcγR polymorphisms can affect the efficacy of monoclonal antibody therapies in IBD (46). The HPO enrichment analysis results of SIRG indicated that abnormal intestinal immunity is a significant phenotype shared by IgAN and IBD. Although the intestinal mucosa is an organ system traditionally associated with IgA (47), the role of IgG and FcγR in the intestinal immunity is crucial.

### TRP Channels May Be a Potential Therapeutic Target for IgAN and IBD

SDEGs and SIRGs are both related to the inflammatory mediator regulation of TRP channels, which are widely distributed in the gastrointestinal tract and not only maintain normal physiological functions of the gastrointestinal tract (48), but also evoke the intestinal inflammation initiated by the local release of immunomodulatory neuropeptides (49). TRP channels are expressed in immune cells and directly contribute to the immune response by affecting the activation of macrophages and CD4+T cells (50, 51). Numerous studies have indicated that TRP channels are mainly involved in the immune pathogenesis of IBD. Inhibition or activation of TRP channels may be an attractive target to ameliorate intestinal inflammation (52). Furthermore, inhibiting the TRP channels related genes TRPC5 could protect podocytes in the kidney and prevent kidney failure (53), TRP channels may be a new target for the prevention and treatment of IgAN and IBD in the future.

## Conclusion

We found that abnormal humoral immunity mediated by Th17 cell may be considered as the shared pathogenic mechanism of IgAN and IBD, and identified novel gene candidates that could be used as biomarkers or potential therapeutic targets.

## Data Availability Statement

The datasets presented in this study can be found in online repositories. The names of the repository/repositories and accession number(s) can be found in the article/**Supplementary Materials**.

## Author Contributions

JQ processed the data, and drafted the paper. CL and XH helped filter datasets for the manuscript. WS, HT and HY helped polish the manuscript. YL was responsible for final review. All authors approved the final version. All authors contributed to the article and approved the submitted version.

## Funding

This work was supported by the the National Natural Science Foundation of China (82170716), the College Science and Technology Innovation Project of Shanxi Education Department, the Key Laboratory Construction Plan Project of Shanxi Provincial Health Commission (2020SYS01) and the Research Project Supported by Shanxi Scholarship Council of China (2020-183).

## Conflict of Interest

The authors declare that the research was conducted in the absence of any commercial or financial relationships that could be construed as a potential conflict of interest.

## Publisher’s Note

All claims expressed in this article are solely those of the authors and do not necessarily represent those of their affiliated organizations, or those of the publisher, the editors and the reviewers. Any product that may be evaluated in this article, or claim that may be made by its manufacturer, is not guaranteed or endorsed by the publisher.

## Supplementary Material

Supplementary Figure 1 The KEGG pathway of Th17 cell differentiation.

Supplementary Figure 2 The KEGG pathway of Th1 and Th2 cell differentiation.
