# Supplementary figures and images for: Differentiation of T Helper 17 Cells May Mediate the Abnormal Humoral Immunity in IgA Nephropathy and Inflammatory Bowel Disease Based on Shared Genetic Effects
Source: Front Immunol. 2022 Jun 13;13:916934. doi: 10.3389/fimmu.2022.916934 (PMC9234173; doi:10.3389/fimmu.2022.916934)

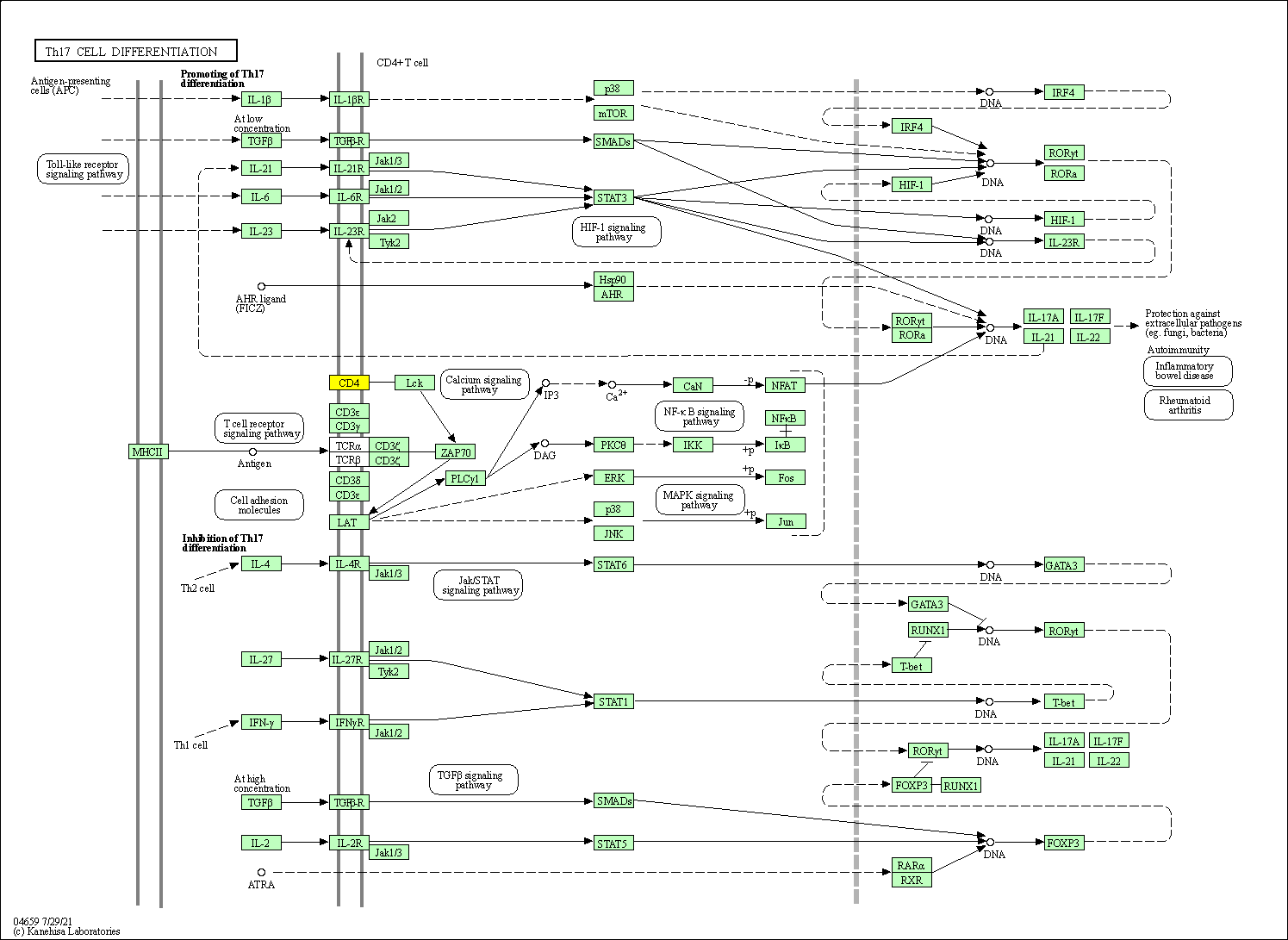

Supplement: Supplementary Figure 1 — The KEGG pathway of Th17 cell differentiation. [file Image_1.jpeg]

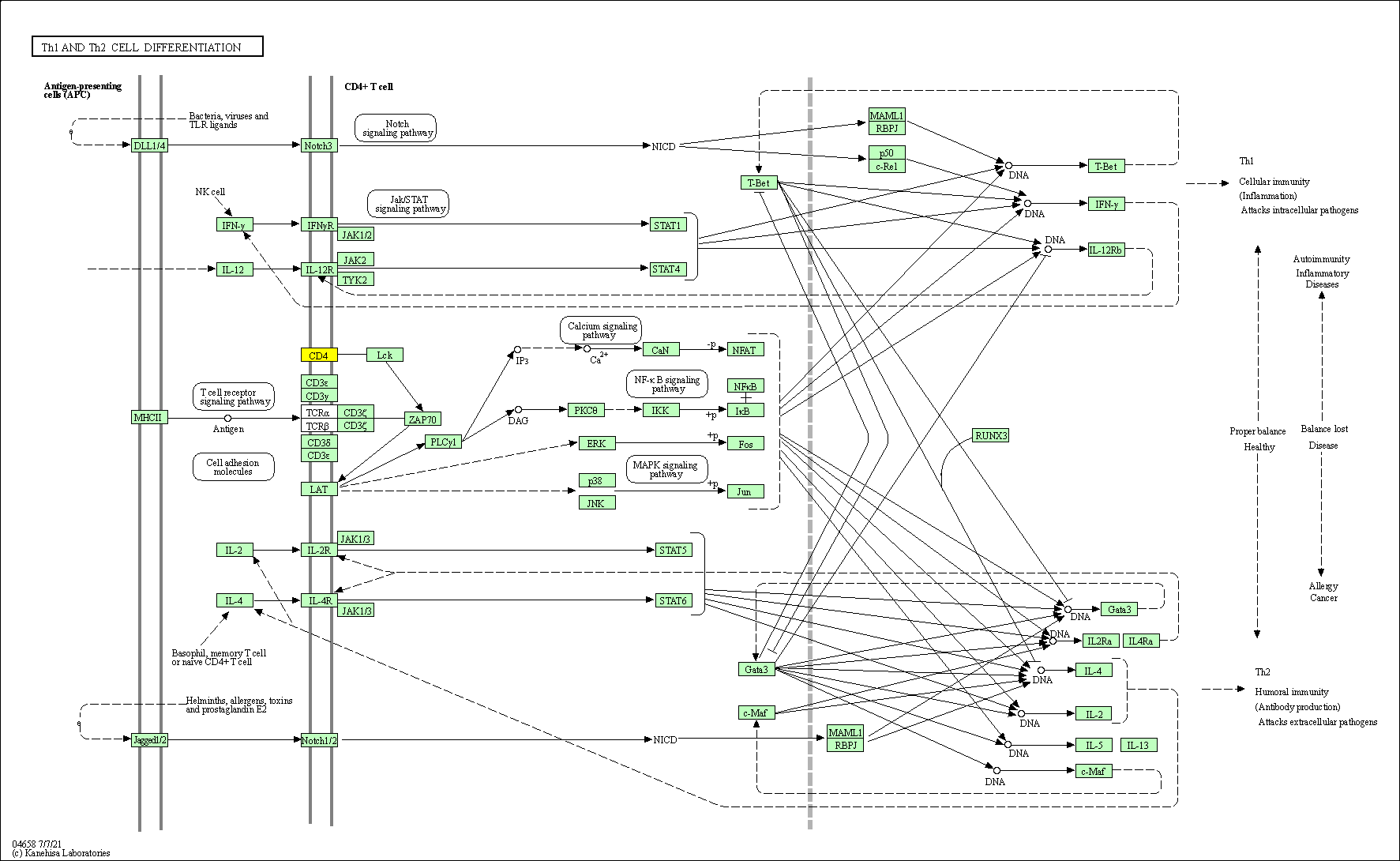

Supplement: Supplementary Figure 2 — The KEGG pathway of Th1 and Th2 cell differentiation. [file Image_2.jpeg]
